# Impact of Pneumonia on Rehabilitation Outcomes: A Large Observational Study

**DOI:** 10.1016/j.arrct.2025.100569

**Published:** 2025-12-10

**Authors:** Van Giap Vu, Thi Linh Tran, Viet Phuong Dao, Xuan Trien Vu, Le Huyen Mai, Thi Thu Ha Bui, Thi Minh Thanh Vu, Dao Quang Do, Thi Thanh Huyen Tran, Dao Vu Do

**Affiliations:** a Respiratory Department, Hanoi Medical University, Hanoi; b Respiratory Center, Bach Mai Hospital, Hanoi; c Department of Rehabilitation, University of Medicine and Pharmacy, Vietnam National University in Hanoi, Hanoi; d Stroke Center, Bach Mai Hospital, Hanoi; e Faculty of Stroke and Cerebrovascular disease, University of Medicine and Pharmacy, Vietnam National University, Hanoi; f Department of Emergency and Critical Care, Hanoi Medical University, Hanoi; g Rehabilitaion Department, E Hospital, Hanoi; h Stroke Center, Phu Tho Provincial General Hospital, Phu Tho; i Neurological Stroke Rehabilitation Department of the Stroke Center, Phu Tho Provincial General Hospital, Phu Tho; j Medical Science, Phenikaa University, Hanoi; k Vinmec HiTech Center, Vinmec Healthcare System, Hanoi; l Rehabilitation Center, Bach Mai Hospital, Hanoi; m Faculty of Clinical Medicine, Hanoi University of Public Health, Hanoi, Vietnam

**Keywords:** Hospital-acquired pneumonia, Poststroke pneumonia, Rehabilitation, Stroke, Stroke-associated pneumonia

## Abstract

**Objective:**

To evaluate the effect of poststroke pneumonia on rehabilitation outcomes in patients with acute stroke.

**Design:**

A prospective observational cohort study.

**Setting:**

Rehabilitation services for patients with stroke across 3 hospitals in a low and middle-income country.

**Participants:**

A total of 922 patients with stroke (N=922) were enrolled (median age: 67y; 63.7% men) and classified into poststroke pneumonia (PSP; n=296) and nonpneumonia (PSNP; n=626) groups. The patients with PSP were further divided into 2 subgroups based on onset timing including stroke-associated pneumonia (SAP; n=157), defined as diagnosis within 7 days of stroke onset, and hospital-acquired pneumonia (HAP; n=139), defined as pneumonia diagnosed after 7 days.

**Interventions:**

Not applicable.

**Main Outcome Measures:**

Primary outcome analysis focused on functional outcomes (modified Rankin Scale [mRS]), hospital length of stay (LOS), and mortality were assessed at discharge, 3 months, and 6 months after stroke. Secondary analysis explored predictors of functional outcomes.

**Results:**

Patients with PSP demonstrated significantly poorer functional outcomes, with higher mRS scores (≥3) at discharge, 3 months, and 6 months (*P*<.001). However, HAP showed stronger association with poor outcomes (odds ratio [OR]≥3) than SAP. Mortality was higher in the PSP group compared with the PSNP group at 3 months (OR=4.98; 95% CI, 2.1-11.7, *P*<.001) and 6 months after stroke (OR=5.4; 95% CI, 2.3-12.6, *P*<.001). HAP also associated with a longer LOS (24.18±7.81d) than SAP (19.5±8.26d; *P*<.001). Stroke severity remained a strong predictor of outcomes in this study.

**Conclusions:**

Poststroke pneumonia worsens rehabilitation outcomes, prolongs LOS, and increases mortality. Early pneumonia prevention and multidisciplinary integrated rehabilitation, aligned with World Health Organization (WHO) standards, are essential to improve stroke recovery and reduce complications in low- and middle-income countries.

## Highlights

• This study compares stroke-associated pneumonia, hospital-acquired pneumonia (HAP), poststroke nonpneumonia groups for poststroke pneumonia (PSP) on rehab outcomes.
• PSP, especially HAP, increased disability, mortality, and hospital stays.
• Highlights need for early pneumonia detection in poststroke care in Vietnam.

Pneumonia is a significant complication after stroke, with incidence rates ranging from 10% to 20%.^1^, ^2^, ^3^, ^4^, ^5^ In acute ischemic stroke, pneumonia affects up to 40% of patients and contributes to a 49% increase in long-term mortality within 1 year after stroke.^6^ Risk factors associated with the development of poststroke pneumonia (PSP) include age, impaired consciousness, atrial fibrillation, dysphagia, and prolonged immobility.^4^^,^^7^^,^^8^ Besides ischemic stroke, recent studies have identified new risk factors including nasogastric tube use and diabetes.^9^ In addition, a study of patients with acute ischemic stroke and dysphagia from Vietnam showed that pneumonia was associated with specific stroke locations, higher stroke severity, tube feeding.^5^

PSP may appear early (within 7 days, as stroke-associated pneumonia [SAP])^10^ or later (as hospital-acquired pneumonia [HAP]).^11^^,^^12^ SAP affects 1.2%-22%^13^ especially up to 40% of patients with stroke—those needing mechanical ventilation.^6^ It is also associated with a 3-fold higher mortality within the first month longer hospital stays, and increased dependency at discharge.^6^^,^^14^^,^^15^ The HAP affects nearly one-third of acute patients with stroke and is the leading cause of death from poststroke medical complications,^16^ with reported mortality rates ranging from 20% to 50%^17^ and 30.6% in one cohort study.^18^ The HAP also prolongs hospital stays by up to 42.9%, reduces mobility, increases dependency at discharge, and contributes to overall health decline.^19^

Interdisciplinary rehabilitation is central to poststroke therapy.^20^ Physical rehabilitation, particularly during the acute phase (first mo after stroke),^21^ is essential for recovery and should begin as early as within 24-48 hours of hospitalization to prevent complications and improve outcomes.^20^^,^^22^^,^^23^ However, PSP can significantly hinder rehabilitation progress. Although pneumonia may not increase long-term mortality, it is associated with higher mortality within the first year after stroke, prolonged hospital length of stay (LOS), and poorer functional outcomes at discharge.^24^, ^25^, ^26^ Risk during the postacute recovery phase is mainly related to patient-specific factors such as dysphagia,^5^ stroke severity measured by National Institutes of Health Stroke Scale (NIHSS),^27^ and nasogastric tube use,^28^ rather than the rehabilitation setting itself.

Despite its clinical importance, there remains a critical gap in published research on the incidence, risk factors, and impact of PSP in low- and middle-income countries (LMICs), where health care resources and rehabilitation services often differ substantially from those in high-income countries. Over 87% of all stroke deaths occurred in LMICs^29^ where, in nations such as Vietnam, stroke is the leading cause of death and disability.^30^ Current evidence is predominantly derived from high-income countries, whereas data from LMICs remain scarce.^31^ These gaps hinder effective guideline development and stroke care improvement on a global scale. By evaluating PSP outcomes in a large cohort (n=922) from major stroke rehabilitation centers in LMICs, this study addresses critical knowledge gaps and provides evidence to inform both national and international stroke policy.

## Methods

### Study design

A prospective observational cohort study.

### Setting

This prospective study recruited 922 patients with stroke from 3 stroke centers in LMICs, including the Stroke Center and Rehabilitation Center at Bach Mai Hospital, the Stroke Center and Rehabilitation Unit at Phu Tho General Hospital, and the Stroke Center and Rehabilitation Department at E Hospital, between February 2022 and April 2024. These centers follow a shared model of stroke care and several characteristics which are provided in supplemental note 1. Data collection and clinical assessments were conducted at baseline, discharge, and 3 and 6 months after stroke, adhering to the Helsinki Declaration and approved by the local Hospital Ethics Committee (Decision no. 4774/QD-BM). Participation was voluntary with written informed consent, and withdrawal carried no health care implications. This study was conducted and reported in accordance with the Strengthening the Reporting of Observational Studies in Epidemiology statement guidelines.^32^^,^^33^

Eligible patients were divided into 2 groups: (1) PSP indicating patients with pneumonia after stroke and (2) poststroke nonpneumonia (PSNP) for patients without pneumonia after stroke. The PSP group was further divided into 2 subgroups: SAP, defined as onset within 7 days of stroke, and HAP, defined as onset after 7 days.^11^^,^^16^

All patients participated in a WHO-guided rehabilitation program.^28^ Patients with PSP received antibiotics prescribed by respiratory specialists and pulmonary rehabilitation according to the British Thoracic Society guidelines.^34^ Follow-up assessments were conducted at discharge, and 3 and 6 months after stroke.

**Fig 1:**
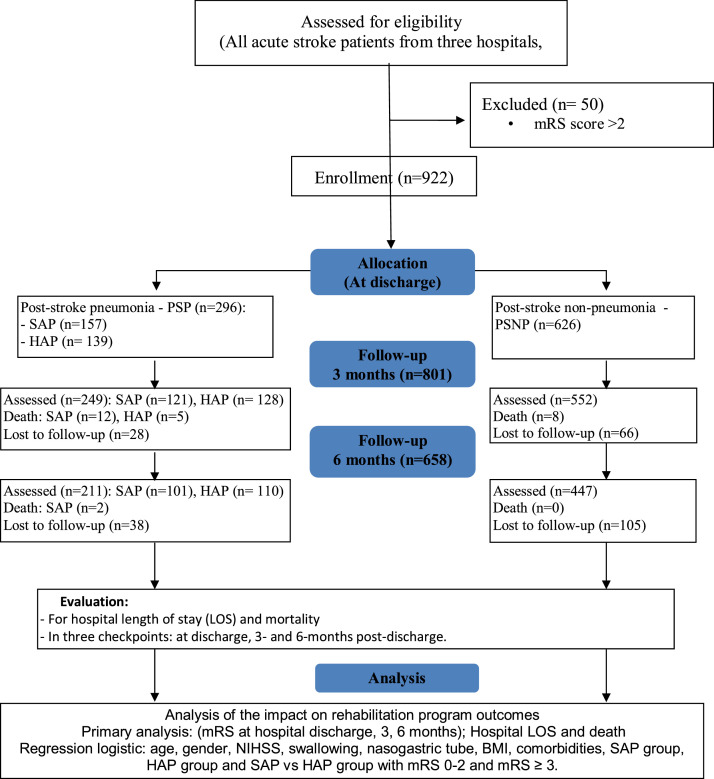
Research study diagram.

This study engaged clinicians and researchers to assess patient conditions and collect data as illustrated in figure 1. Information was obtained from interviews, medical records, vital signs, laboratory tests, imaging results, and standardized assessments including NIHSS and modified Rankin Scale (mRS). All data were entered into an electronic database for analysis, focusing on baseline characteristics and outcomes.

### Participants

Eligible participants were selected based on the inclusion and exclusion criteria in table 1. Briefly the patients were adults (≥18y) with a confirmed diagnosis of acute stroke according to WHO criteria, verified by computed tomography scan or magnetic resonance imaging.^12^^,^^21^ They were assigned to rehabilitation therapy and provided written informed consent. The diagnosis classified the patients into 2 groups: (1) PSP indicating patients with pneumonia after stroke and (2) PSNP for patients without pneumonia after stroke. Within the PSP group, patients were divided into 2 subgroups: early-onset SAP, if they get PSP within 7 days after stroke, and HAP, if they get PSP after 7 days. Exclusion criteria for the patients included: (i) transient ischemic attack, (ii) pneumonia before the acute stroke, (iii) severe prestroke (mRS ≥3),^35^^,^^36^ (iv) refusal to participation, (v) or unstable physiological variables (systolic blood pressure of <110 mmHg or >220 mmHg; oxygen saturation of <92% with supplementation; resting heart rate of <40 or >110 beats per minute; body temperature >38.5 °C).

**Table 1:** Inclusion and exclusion criteria for the eligible participants in this study.

Inclusion Criteria	Exclusion Criteria
• Age ≥18 y • Acute stroke confirmed by WHO criteria and CT/MRI • Diagnosis of SAP or HAP not requiring mechanical ventilation (PSP group), or no pneumonia during hospitalization (PSNP—control group) • Provided a written informed consent • Assigned to rehabilitation therapy	• Transient ischemic attack (TIA) • Pneumonia before acute stroke • Severe prestroke disability (mRS≥3) • Declined participation • Unstable physiological variables: SBP <110 or >220 mmHg; SpO_2_ <92% with oxygen; heart rate <40 or >110 bpm; body temperature >38.5 °C

### Variables

#### Baseline characteristics of participants

The baseline characteristics of participants included demographic data (age, sex), clinical, and functional variables**.** Medical history data encompassed the presence of diabetes, atrial fibrillation, prior stroke, and dependency before stroke. Clinical characteristics were evaluated using the NIHSS score on admission, categorized as mild (1-4), moderate (5-15), and severe (>15).^37^ Additional variables included time to rehabilitation (≤7d or >7d from stroke onset)^4^ and LOS from admission to discharge and disorders of consciousness measured by Glasgow coma scale with a score ≤14 indicating impaired consciousness.^38^ Swallowing function was evaluated based on nasogastric tube placement. Stroke classification was ischemic or hemorrhagic stroke. The subtypes of ischemic stroke^39^^,^^40^ included total anterior circulation infarct, partial anterior circulation infarct, posterior circulation infarct, and lacunar infarct. The cerebral hemorrhage subtype was intracerebral hemorrhage and subarachnoid hemorrhage. Finally, functional outcome was assessed using the mRS with score of 0-2 defined as good outcomes and scores ≥3 defined as poor outcomes.

The primary outcome measures of the study were mortality, hospital (LOS), and functional outcomes assessed by mRS scores at discharge, 3 and 6 months after stroke onset. Secondary analyses aimed to identify predictors of functional outcomes, focusing on variables such as age, sex, NIHSS, swallowing ability, nasogastric tube placement, body mass index, and comorbidities.

### Data sources/measurement

We included all in patients with acute stroke who met the predefined inclusion criteria. Eligible patients were consecutively screened and enrolled after obtaining ethical approval and informed consent. This sampling approach was used because the inpatient setting provided ready access to the target population and enabled systematic data collection within routine clinical care.

### Statistical methods

In this study, we used convenient sampling approach to selected nonrandom samples that were based on their availability regardless of age, stroke type, and clinical condition throughout the study period.

The data were analyzed using SPSS version 20.^a^ Student *t*test and Mann-Whitney *U* test were applied for continuous variables, depending on distribution. For categorical variables (eg, age group, NIHSS categories), the chi-square test was applied to compare groups. When expected cases were less than 5, the Fisher exact test was applied instead. Logistic regression analysis was performed to evaluate the effects of independent variables on functional outcomes (mRS scores: 0-2=good, >2=poor outcome). Statistical significance was defined as *P*<.05.

## Results

### Participants

A total of 922 participants were included, with Bach Mai Hospital recruiting 444 (48.2%), Phu Tho General Hospital 422 (45.8%), and E Hospital 56 (6.1%) (table 2). All hospitals implemented early rehabilitation in accordance with WHO guidelines,^28^ from 24 hours after stroke.

**Table 2:** Patient distribution in 3 hospitals.

Hospital	No. of Patients	Percent
Bach Mai Hospital	444	48.2
Phu Tho General Hospital	422	45.8
E Hospital	56	6.1
Total	922	100.0

### Comparison between the pneumonia and nonpneumonia stroke groups

Table 3 outlines baseline characteristics of patients with stroke. The median age of the cohort was 67 years (IQR 58-74). Patients in the PSP group were older than those in the PSNP group (*P*=.009). Pneumonia was more common in older patients (>75y; *P*=.016). Sex distribution was predominantly men (63.7%) with no significant group differences (*P*=.337). Risk factors such as atrial fibrillation, diabetes, premorbid mRS, stroke history, and cerebral hemorrhage showed no significant variance (*P*>.05).

**Table 3:** Baseline characteristics of patients with stroke with and without pneumonia.

Baseline characteristics	All (n=922)	PSP (n=296)	PSNP (n=626)	*P* Value
Age (y), median (IQR)	67 (58-74)	68 (60-76)	66 (57-74)	.009†
≤75 y, n (%)	720 (78.09)	217 (73.31)	503 (80.35)	.016
>75 y, n (%)	202 (21.91)	79 (26.69)	123 (19.65)	
Sex, n (%)				
Male	587 (63.67)	195 (65.88)	392 (62.62)	.337
Female	335 (36.33)	101 (34.12)	234 (37.38)	
Risk factors, n (%)				
Atrial fibrillation	52 (5.64)	22 (7.43)	30 (4.79)	.105
Diabetes mellitus	228 (24.73)	64 (21.62)	164 (26.20)	.133
Premorbid mRS, n (%)				
0	837 (90.78)	265 (89.53)	572 (91.37)	.3
1	64 (6.94)	21 (7.09)	43 (6.87)	
2	21 (2.28)	10 (3.38)	11 (1.76)	
Stroke history, n (%)	160 (17.35)	56 (18.92)	104 (16.61)	.388
Time to rehabilitation (d), median (IQR)	4 (3-8)	5 (3-10)	4 (3-7)	<.001†
Early rehab (≤7d), n (%)	681 (73.86)	195 (65.88)	486 (77.64)	<.001
Delayed rehab (>7d), n (%)	241 (26.14)	101 (34.12)	140 (22.36)	
NIHSS score, median (IQR)	8 (5-12)	11 (8-15)	7 (4-10)	<.001†
Mild (1-4), n (%)	218 (23.64)	31 (10.47)	187 (29.87)	<.001
Moderate (5-15), n (%)	621 (67.35)	197 (66.55)	424 (67.73)	
Severe (≥16), n (%)	83 (9.01)	68 (22.97)	15 (2.40)	
Glasgow score, median (IQR)	15 (15-15)	14 (13-15)	15 (15-15)	<.001†
Disorders of consciousness				
No (15), n (%)	729 (79.07)	147 (49.66)	582 (92.97)	<.001
Yes (≤14), n (%)	193 (20.93)	149 (50.34)	44 (7.03)	
Stroke type, n (%)				
Cerebral hemorrhage	188 (20.39)	90 (30.41)	98 (15.65)	<.001
Ischemic stroke	734 (79.61)	206 (69.59)	528 (84.35)	
Stroke ischemic type, n (%)				
Total ACI	92 (12.53)	30 (14.56)	62 (11.74)	.356
Partial ACI	503 (68.53)	137 (66.50)	366 (69.32)	
Posterior circulation infarct	128 (17.43)	38 (18.45)	90 (17.05)	
Lacunar infarct	11 (1.50)	1 (0.49)	10 (1.89)	
Cerebral hemorrhage, n (%)				
Intracerebral	157 (83.51)	74 (82.22)	83 (84.69)	.648
Subarachnoid	31 (16.49)	16 (17.78)	15 (15.31)	
Nasogastric tube, n (%)	157 (83.51)	74 (82.22)	83 (84.69)	<.001
Hospital LOS (d) Mean (IQR)	18 (12-25)	21 (14-28)	16 (11-23)	<.001†

Abbreviation: ACI, anterior circulation infarct.

*P*: χ^2^ test.

† *P*: Mann-Whitney *U* test.

Among all patients, 681 patients (73.9%) received early rehabilitation (≤7d), whereas 241 (26.1%) had delayed rehabilitation (>7d). The pneumonia group had a 1-day median delay (5 vs 4d, *P*<.001) and had fewer patients starting early rehabilitation (65.9% vs 77.6%, *P*<.001).

Patients with pneumonia had significantly higher NIHSS scores, reflecting more severe strokes (median NIHSS 11 vs 7, *P*<.001). Mild strokes were less frequent in patients with pneumonia, whereas moderate and severe strokes were more common. Pneumonia was also associated with a higher incidence of consciousness disorders (*P*<.001).

Ischemic strokes were more prevalent (*P*<.001), but no correlation was found between the type of ischemic stroke and pneumonia. Patients with pneumonia had higher rates of nasogastric tube usage (*P*<.001) and longer hospital stays (21.99±8.31 vs 17.85±7.95 days, *P*<.001).

### Impacts of pneumonia on stroke outcomes over a 6-month follow-up period

**Fig 2:**
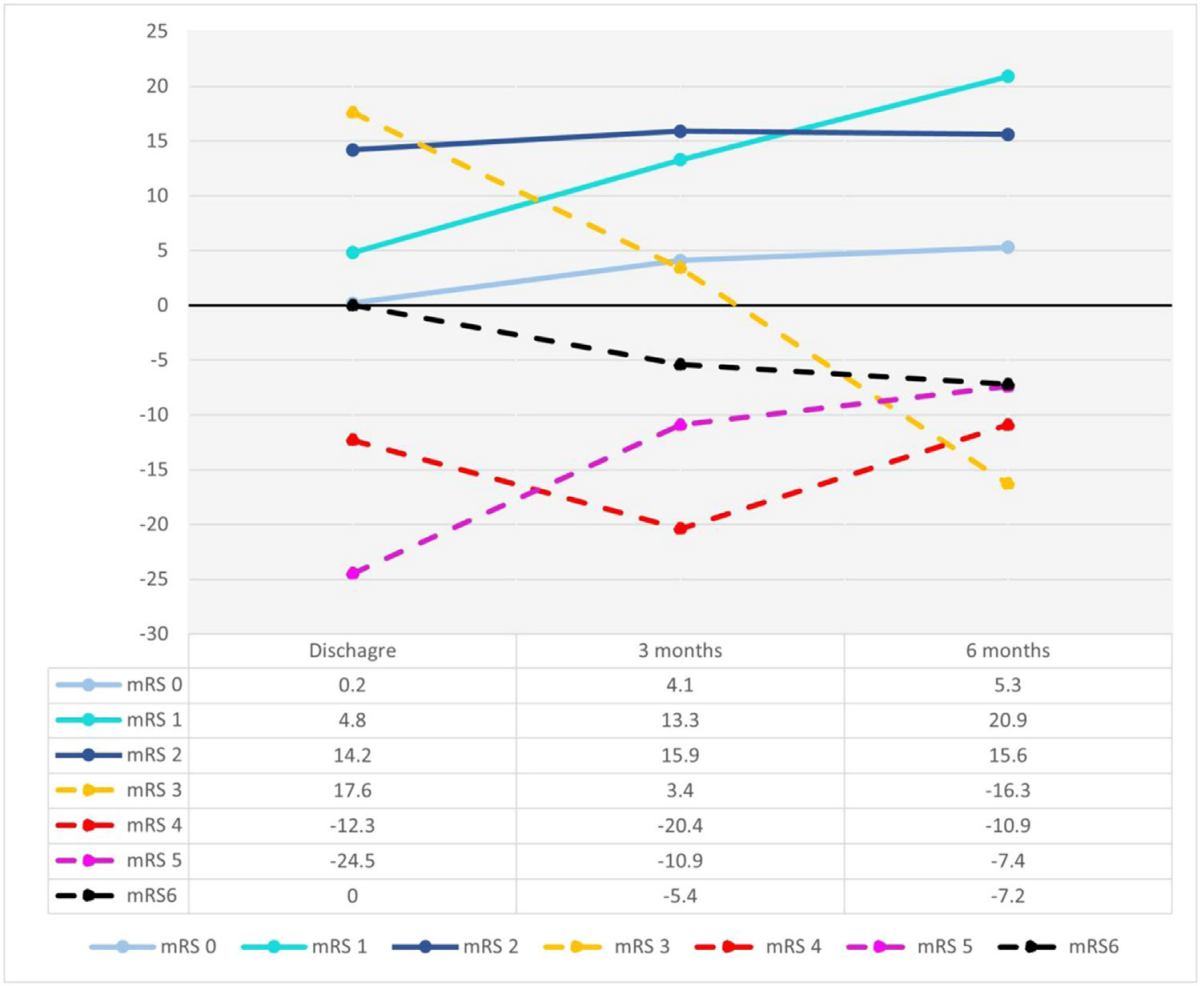
Difference of patient proportion in PSNP (nonpneumonia) and PSP (pneumonia) groups. The y-axis shows the difference in patient proportions, with positive values indicating a higher proportion in the PSNP group. Differences are calculated for each mRS score at each time point (x-axis). Functional outcomes are categorized as good (mRS 0-2, solid lines) and poor (mRS 3-6, dashed lines).

Comparison of mRS scores (0-6) at discharge, 3 months, and 6 months revealed better outcomes in the PSNP group, with improvements over time (fig 2). In contrast, the PSP group showed an increasing rate of poor outcomes (mRS 3-6) long with higher mortality rates and longer hospital stays. The PSNP group showed declining rates of poor outcomes (mRS 3) from 33.55% at discharge to 18.34% at 6 months, whereas the PSP group saw an increase from 15.88% to 34.6%. Very poor outcomes (mRS 4-5) and mortality (mRS 6) were significantly higher in the PSP group across all time points (mRS 4: 7.6% vs 18.5%, <0.001; mRS 5: 1.57% vs 9.0%, *P*<.001, respectively) (supplemental table S1, available online only at http://www.archives-pmr.org/). Mortality was consistently higher in the PSP group at both 3 months and 6 months (supplemental table S2, available online only at http://www.archives-pmr.org/).

### Mortality and hospital length of stay between patient groups

Overall mortality was low (<3%) across groups (supplemental table S2), but rates were significantly higher in the PSP group compared with PSNP (*P*<.05, table 4). Specifically, mortality in the PSP group was higher than in the PSNP group at both 3 months (odds ratio [OR]=4.98; 95% CI, 2.1-11.7; *P*<.001) and 6 months after stroke (OR=5.4; 95% CI, 2.3-12.6; *P*<.001). Although these 95% CIs are relatively wide, their lower bounds remain above 2.0, well above 1.0, supporting their significance and direction. Of note, the *P* values and the original CIs were calculated using the log ORs. Because ORs are on an exponential scale, their 95% CIs naturally appear wider.

**Table 4:** Clinical outcomes between patient groups including PSP, HAP, SAP, and PSNP.

	Primary Outcome			Functional Outcome (mRS)		
	Mortality at 3 mo	Mortality at 6 mo	LOS	Discharge	3 mo	6 mo
PSP vs PSNP						
OR (95% CI)	4.98 (2.12-11.71)	5.43 (2.34-12.62)		4.49 (2.81-7.19)	4.75 (3.31-6.81)	5.93 (4.14-8.51)
*P* value	<.001	<.001	<.001†	<.001	<.001	<.001
SAP vs PSNP						
OR (95% CI)	6.13 (2.37-15.87)	7.40 (2.94-18.63)		5.89 (2.94-11.81)	4.84 (2.96-7.91)	6.29 (3.89-10.17)
*P* value	<.001	<.001	.078†	<.001	<.001	<.001
HAP vs PSNP						
OR (95% CI)	3.93 (1.40-11.06)	3.79 (1.32-10.52)		3.20 (1.79-5.71)	4.66 (2.90-7.49)	5.63 (3.57-8.88)
*P* value	.005	.008	<.001†	<.001	<.001	<.001
SAP vs HAP						
OR (95% CI)	1.56 (0.57-4.24)	1.98 (0.75-5.26)		1.84 (0.77-4.41)	1.04 (0.55-1.97)	1.12 (0.62-2.03)
*P* value	.382	.162	<.001†	.164	.908	.715

*P*: χ^2^ test.

† *P*: Mann-Whitney *U* test.

No significant difference in mortality was observed between SAP and HAP groups (*P*=.382 for 3mo: *P*=.162 for 6mo). The PSP group had longer hospital LOS (21.99±8.31d) compared with the PSNP group (17.85±7.95d) (*P*<.001). Among patients with PSP, SAP had shorter stays than HAP (*P*<.001) (table 4: supplemental tables S2-S5 [available online only at http://www.archives-pmr.org/]).

### Functional outcomes across patient groups

Table 4 highlights mRS-based functional outcomes at discharge, 3 months, and 6 months after stroke (supplemental table S6, available online only at http://www.archives-pmr.org/). Patients with PSP showed a significantly higher proportion of patients with poorer outcomes (mRS≥3) compared with patients with PSNP (*P*<.001). The SAP subgroup showed the worst outcomes, with OR of 5.89 at discharge (95% CI, 2.94-11.81), whereas the HAP subgroup had OR of 3.2 (95% CI, 1.19-5.71). Functional differences between SAP and HAP were not statistically significant (*P*=.164). Full analyses are provided in supplemental tables S6-S8.

### Multivariable analysis of factors influencing functional outcomes in acute patients with stroke

**Fig 3:**
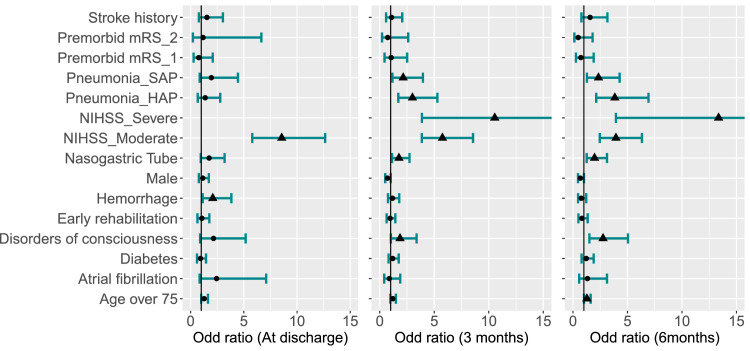
Multivariable analysis of factors associated with functional outcomes (mRS) at discharge, 3 months and 6 months. Each variable’s odds ratio is shown as a dot (*P*>.05) or triangle (*P*≤.05), with a line indicating the 95% CI. Baselines: Premorbid mRS=0 (fully recovered), Pneumonia=PSNP (no pneumonia), and HIHSS=mild stroke.

Figure 3 highlights logistic regression analysis findings on factors affecting acute stroke outcomes. At discharge, cerebral hemorrhage and moderate NIIHSS scores significantly increased the likelihood of poor outcomes. Patients with cerebral hemorrhage had more than twice the risk of poor outcomes compared with those with ischemic stroke (95% CI, 1.13-3.83, *P*=.019). Moderate NIHSS scores were associated with 8.56-fold increased risk of poor outcomes compared with those with mild stroke (95% CI, 5.79-12.64, *P*<.001). Pneumonia had limited impact at discharge but became more prominent at follow-ups. The details of the analysis are provided in supplemental table S9 (available online only at http://www.archives-pmr.org/).

Patients with SAP showed a 2.15-fold increased risk of poor outcomes at 3 months compared with patients with no pneumonia (95% CI, 1.16-3.96, *P*=.014) and a 2.34-fold increased risk at 6 months (95% CI, 1.28-4.28, *P*=.006). HAP is even more strongly associated with poor outcomes, with ORs of 3.00 at 3 months (95% CI, 1.70-5.29, *P*<.001) and 3.84 at 6 months (95% CI, 3.13-6.93, *P*<.001) (supplemental tables S10 and S11 [available online only at http://www.archives-pmr.org/]).

Moderate and severe NIHSS scores were consistently linked to poor outcomes. Patients with moderate stroke severity had a 5.57-fold increased risk of poor outcomes at 3 months compared with those with mild stroke cases (95% CI, 3.86-8.56; *P*<.001) and 3.94-fold higher risk at 6 months (95% CI, 2.46-6.33; *P*<.001). Severe cases showed a significantly increased risk of 10.55-fold at 3 months compared with patients without a nasogastric tube (95% CI, 3.87-28.76; *P*<.001) and 13.36-fold at 6 months (95% CI, 3.94-45.37; *P*<.001) (supplemental table S10).

Nasogastric tube placement because of swallowing disorders increased the risk of poor outcomes: 1.76-times higher at 3 months compared with patients without a nasogastric tube (95% CI, 1.13-2.73; *P*=.012) and 1.98 times higher at 6 months (95% CI, 1.26-3.12; *P*=.003). Individuals over 75 had a 1.28-fold higher risk of poor outcomes at 6 months compared with patients aged 75 years and below (95% CI, 1.01-1.62; *P*=.04). Disorders of consciousness at admission increased the likelihood of recovery, with a 1.86 times at 3 months compared with patients with normal consciousness (95% CI, 1.02-3.38; *P*=.043) and a 2.76 times higher risk at 6 months (95% CI, 1.51-5.05; *P*=.001) (supplemental table S12, available online only at http://www.archives-pmr.org/).

## Discussion

This study highlights the substantial impact of PSP on rehabilitation outcomes, demonstrating its association with increased disability, mortality, and prolonged hospital stays. Our study findings align with prior studies^11^^,^^41^ illustrating that PSP consistently worsens patients’ trajectories across all assessed time points. Notably, the Vietnamese cohort in this study exhibited a higher rate of severe disability (49.8% with mRS≥4) compared with previously reported figures (32.7%),^13^ underscoring the persistent burden of PSP in poststroke populations. Despite functional improvements observed in both PSP and PSNP groups by 6 months, patients with PSP faced higher rates of severe disability (mRS≥3) and mortality (mRS=6) at 6 months compared with patients with PSNP, underscoring the long-term impact of pneumonia on stroke recovery and the need for effective prevention and management strategies.

Pneumonia also significantly affected mortality and hospital LOS with increased mortality evidence at both 3 months and 6 months. This finding is in line with recent research,^41^ which reported higher in-hospital mortality rates in patients with PSP and poorer functional outcomes at 90 days after stroke. Other studies^24^^,^^42^ similarly indicated that SAP increases mortality, LOS, and poor functional outcomes at discharge. Although both SAP and HAP raised mortality rates, no significant differences were found, indicating equal clinical importance.

Our multivariable analysis enhances existing knowledge by elucidating the independent roles of pneumonia and stroke severity in shaping long-term functional outcomes. Although previous studies^43^^,^^44^ primarily focused on outcomes at 3 months, our study offers a broader perspective on the 6-month trajectory. Specifically, patients with moderate NIHSS scores showed a gradual reduction in risk over time, whereas those with severe NIHSS scores remained at consistently elevated risk throughout follow-up.

Interestingly, the impact of pneumonia (SAP and HAP) became more pronounced over time, despite having no significant effect at discharge. Both SAP and HAP showed increasing odds of poor functional outcomes at 6 months, with HAP emerging as a stronger predictor of long-term disability compared with SAP. This suggests a more severe and sustained detrimental effect of HAP. This finding emphasizes the importance of differentiating between PSP subtypes when assessing rehabilitation prognosis and tailoring clinical intervention.

In addition, disorders of consciousness were clearly linked to an increased risk of poor outcomes, consistent with previous studies highlighting the rehabilitation challenges posed by impaired awareness.^45^ Among older patients (aged 75 and above), a modest but significant risk of poor outcomes was observed, reflecting the known vulnerabilities of this population.

### Study limitations

Despite yielding valuable insights, the study had limitations, including the absence of follow-up beyond 6 months, missing later-stage data, and the omission of the Barthel Index for daily living functions assessment. Future research should focus on longer-term outcomes, use broader recovery measures, and compare SAP and HAP to enhance tailored interventions. The relatively small sample size may reduce the precision of regression estimates, and thus, findings should be interpreted cautiously.

## Conclusions

This study stands out as one of the few large-scale investigations into PSP conducted at major stroke centers in Vietnam. By drawing attention to the significant adverse effects of PSP, including increased disability, mortality, and extended hospital stays, the study underscores the profound implications for rehabilitation outcomes. The progressive and enduring impact of pneumonia, especially HAP, highlights the critical importance of early detection and intervention, robust preventive strategies, and a targeted approach to poststroke care to optimize long-term recovery and reduce the burden on families, caretakers, and health care systems.

## Supplier

a. SPSS, version 20; IBM.

## Disclosure

The investigators have no financial or nonfinancial disclosures to make in relation to this project.

## Appendix Supplementary materials

Image, application 1

## Footnotes

Supplementary material associated with this article can be found, in the online version, at doi:10.1016/j.arrct.2025.100569.
