# Supplementary material for: Impact of Pneumonia on Rehabilitation Outcomes: A Large Observational Study
Source: Arch Rehabil Res Clin Transl. 2025 Dec 10;8(1):100569. doi: 10.1016/j.arrct.2025.100569 (PMC12988551; doi:10.1016/j.arrct.2025.100569)
Supplement: Supplementary file 1 [file mmc1.docx]

**Supplementary documents for**

Impact of pneumonia on rehabilitation outcomes: A large observational study

# SUPPLEMENTARY MATERIAL

**Supplementary Note 1:** Shared model and characteristics between the three hospitals.

- Models of stroke unit care:
- Intensive care unit for the first 24-48 hours (depending on the condition)
- Acute stroke ward: acute unit in a discrete ward (usually discharged within seven days)
- Comprehensive stroke unit care: combined acute and rehabilitation unit in a discrete ward
- Stroke rehabilitation unit: a discrete rehabilitation unit for stroke patients who are transferred from acute care 1–2 weeks post-stroke
- Mixed rehabilitation ward: rehabilitation provided on a ward managing a general caseload.

Characteristics of the three hospitals:

- Location in a geographically discrete unit
- Comprehensive assessments
- A coordinated multidisciplinary team
- Early mobilization and avoidance of bed-rest
- Staff with a special interest in the management of stroke, and access to ongoing professional education and training
- Clear communication, with regular team meetings to discuss management (including discharge planning) and other meetings as needed (e.g. family conferences)
- Active encouragement of stroke survivors and their carers/ families to be involved in the rehabilitation process.

# SUPPLEMENTRY TABLE LEGENDS*

**Table S1**. Outcomes at discharge, 3 and 6 months

| Features | PSP | | PSNP | OR (95% CI) | P value |
| --- | --- | --- | --- | --- | --- |
| **Discharge** | **n = 296** | | **n = 626** |  |  |
| mRS score of ≥3 | 274(92.57) | | 461(73.64) | 4.49(2.81-7.19) | < 0.001 |
| mRS score of 0–2 | 22(7.43) | | 165(26.36) |  |  |
| **Secondary (**mRS category) | | |  |  |  |
|  |  | |  |  |  |
| 0 | 0(0.00) | | 1(0.16) |  | < 0.001 |
| 1 | 3(1.01) | | 36(5.75) |  |  |
| 2 | 19(6.42) | | 128(20.45) |  |  |
| 3 | 47(15.88) | | 210(33.55) |  |  |
| 4 | 141(47.64) | | 222(35.46) |  |  |
| 5 | 86(29.05) | | 29(4.63) |  |  |
| **Primary (3 months)** | **n = 249** | | **n = 552** |  |  |
| mRS score of ≥3 | 203(81.53) | | 266(48.19) | 4.75(3.31-6.81) | < 0.001 |
| mRS score of 0–2 | 46(18.47) | | 286(51.81) |  |  |
| **Secondary (**mRS category) | | | | | |
|  | |  |  |  |  |
| 0 | | 1(0.40) | 25(4.53) |  | < 0.001 |
| 1 | | 16(6.43) | 109(19.75) |  |  |
| 2 | | 29(11.65) | 152(27.54) |  |  |
| 3 | | 62(24.90) | 156(28.26) |  |  |
| 4 | | 92(36.95) | 91(16.49) |  |  |
| 5 | | 32(12.85) | 11(1.99) |  |  |
| 6 | | 17(6.83) | 8(1.45) |  |  |
| **Primary (6 months)** | | **n = 211** | **n = 447** |  |  |
| mRS score of ≥3 | | 150(71.09) | 131(29.31) | 5.93(4.14-8.51) | < 0.001 |
| mRS score of 0–2 | | 61(28.91) | 316(70.69) |  |  |
| **Secondary (**mRS category) | | | | | |
| 0 | | 4(1.90) | 32(7.16) |  | < 0.001 |
| 1 | | 21(9.95) | 138(30.87) |  |  |
| 2 | | 36(17.06) | 146(32.66) |  |  |
| 3 | | 73(34.60) | 82(18.34) |  |  |
| 4 | | 39(18.48) | 34(7.61) |  |  |
| 5 | | 19(9.00) | 7(1.57) |  |  |
| 6 | | 19(9.00) | 8(1.79) |  |  |

Abbreviation: PSP: Post Stroke Pneumonia; PSNP: Post Stroke Non-Pneumonia; mRS: Modified Rankin Scale.

**Table S2**. Death and Hospital LOS between the two groups PSP and PSNP

| Features | PSP | | PSNP | | OR (95% CI) | P value |
| --- | --- | --- | --- | --- | --- | --- |
|  | No. at risk | No. of deaths (%) | No. at risk | No. of deaths (%) |  |  |
| 3-month (n=801) | 249 | 17(2.12) | 552 | 8(1.00) | 4.98(2.12-11.71) | < 0.001 |
| 6-month (n=658) | 211 | 19(2.89) | 447 | 8(1.22) | 5.43(2.34-12.62) | < 0.001 |
| Hospital LOS (day: mean) | 21.99 ± 8.31 | | 17.85 ± 7.95 | |  | < 0.001 |

Abbreviation: LOS: length of stay.

**Table S3**. Death and hospital LOS between the two groups SAP and PSNP

| Features | SAP | | PSNP | | OR (95% CI) | P value |
| --- | --- | --- | --- | --- | --- | --- |
|  | No. at risk | No. of deaths (%) | No. at risk | No. of deaths (%) |  |  |
| 3-month (n=801) | 121 | 10(1.48) | 552 | 8(1.19) | 6.13(2.37-15.87) | < 0.001 |
| 6-month (n=658) | 101 | 12(2.19) | 447 | 8(1.45) | 7.4(2.94-18.63) | < 0.001 |
| Hospital LOS (day: mean) | 19.5 ± 8.26 | | 17.85 ± 7.95 | |  | 0.078 |

Abbreviation: SAP: Stroke-Associated Pneumonia

**Table S4**. Death and hospital LOS between the two groups HAP and PSNP

| Features | HAP | | PSNP | | OR (95% CI) | P value |
| --- | --- | --- | --- | --- | --- | --- |
|  | No. at risk | No. of deaths (%) | No. at risk | No. of deaths (%) |  |  |
| 3-month (n=801) | 128 | 7(1.03) | 552 | 8(1.18) | 3.93(1.40-11.06) | 0.005 |
| 6-month (n=658) | 110 | 7(1.26) | 447 | 8(1.44) | 3.79(1.32-10.52) | 0.008 |
| Hospital LOS (day: mean) | 24.18 ± 7.81 | | 17.85 ± 7.95 | |  | < 0.001 |

Abbreviation: HAP: Hospital-Acquired Pneumonia

**Table S5**. Death and hospital LOS between the two groups SAP and HAP

| Features | HAP | | SAP | | OR (95% CI) | P value |
| --- | --- | --- | --- | --- | --- | --- |
|  | No. at risk | No. of deaths (%) | No. at risk | No. of deaths (%) |  |  |
| 3-month (n=801) | 128 | 7(2.81) | 121 | 10(4.02) | 1.56(0.57-4.24) | 0.382 |
| 6-month (n=658) | 110 | 7(3.32) | 101 | 12(5.69) | 1.98 (0.75-5.26) | 0.162 |
| Hospital LOS (day: mean) | 24.18 ± 7.81 | | 19.5 ± 8.26 | | 19.5 ± 8.26 | < 0.001 |

**Table S6**. Analysis subgroup between SAP and PSNP

|  | Total | | SAP | PSNP | OR (95% CI) | P value |
| --- | --- | --- | --- | --- | --- | --- |
| **Discharge** | | **n = 922** | **n = 157** | **n =626** |  |  |
| mRS score of ≥3 | | 734(79.61) | 148(94.27) | 461(73.64) | **5.89 (2.94-11.81)** | < 0.001 |
| mRS score of 0–2 | | 188(20.39) | 9(5.73) | 165(26.36 |  |  |
| **Secondary** | |  |  |  |  |  |
| mRS category | |  |  |  |  |  |
| 0 | | 1(0.11) | 0(0.00) | 1(0.16) |  | < 0.001 |
| 1 | | 39(4.23) | 2(1.27) | 36(5.75) |  |  |
| 2 | | 148(16.05) | 7(4.46) | 128(20.45) |  |  |
| 3 | | 257(27.87) | 19(12.10) | 210(33.55) |  |  |
| 4 | | 362(39.26) | 72(45.86) | 222(35.46) |  |  |
| 5 | | 115(12.47) | 57(36.31) | 29(4.63) |  |  |
| **Primary (3 months)** | | **n = 801** | **n = 121** | **n = 552** |  |  |
| mRS score of ≥3 | | 469(58.55) | 99(81.82) | 266(48.19) | **4.84(2.96-7.91)** | < 0.001 |
| mRS score of 0–2 | | 332(41.45) | 22(18.18) | 286(51.81) |  |  |
| **Secondary (**mRS category) | | | | | | |
| 0 | | 26(3.25) | 0(0.00) | 25(4.53) |  | < 0.001 |
| 1 | | 125(15.61) | 7(5.79) | 109(19.75) |  |  |
| 2 | | 181(22.60) | 15(12.40) | 152(27.54) |  |  |
| 3 | | 218(27.22) | 19(15.70) | 156(28.26) |  |  |
| 4 | | 183(22.85) | 48(39.67) | 91(16.49) |  |  |
| 5 | | 43(5.37) | 22(18.18) | 11(1.99) |  |  |
| 6 | | 25(3.12) | 10(8.26) | 8(1.45) |  |  |
| **Primary (6 months)** | | **n =658** | **n = 101** | **n = 447** |  |  |
| mRS score of ≥3 | | 281(42.71) | 73(72.28) | 131(29.31) | **6.29 (3.89-10.17**) | < 0.001 |
| mRS score of 0–2 | | 377(57.29) | 28(27.72) | 316(70.69) |  |  |
| **Secondary (**mRS category) | | | | | | |
| 0 | | 36(5.47) | 2(1.98) | 32(7.16) |  | < 0.001 |
| 1 | | 159(24.16) | 11(10.89) | 138(30.87) |  |  |
| 2 | | 182(27.66) | 15(14.85) | 146(32.66) |  |  |
| 3 | | 155(23.56) | 23(22.77) | 82(18.34) |  |  |
| 4 | | 73(11.09) | 25(24.75) | 34(7.61) |  |  |
| 5 | | 26(3.95) | 13(12.87) | 7(1.57) |  |  |
| 6 | | 27(4.10) | 12(11.88) | 8(1.79) |  |  |

**Table S7**. Analysis subgroup between HAP and PSNP

|  | Total | HAP | | PSNP | | OR (95% CI) | | P value |  |
| --- | --- | --- | --- | --- | --- | --- | --- | --- | --- |
| **Discharge** | **922(100)** | **139(15.08)** | | **626(67.9)** | |  | |  |  |
| mRS score of ≥3 | 734(79.61) | | 125(89.93) | | 461(73.64) | | 3.20(1.79-5.71) | < 0.001 | |
| mRS score of 0–2 | 188(20.39) | 14(10.07) | | **165(26.36)** | |  | |  |  |
| **Secondary** (mRS category) | | | | | | | | |  |
| 0 | 1(0.11) | 0(0.00) | | 1(0.16) | |  | | < 0.001 |  |
| 1 | 39(4.23) | 1(0.72) | | 36(5.75) | |  | |  |  |
| 2 | 148(16.05) | 13(9.35) | | 128(20.45) | |  | |  |  |
| 3 | 257(27.87) | 28(20.14) | | 210(33.55) | |  | |  |  |
| 4 | 362(39.26) | 68(48.92) | | 222(35.46) | |  | |  |  |
| 5 | 115(12.47) | 29(20.86) | | 29(4.63) | |  | |  |  |
| **Primary (3 months)** | **n = 801** | **n = 128** | | **n = 552** | |  | |  |  |
| mRS score of ≥3 | 469(58.55) | 104(81.25) | | 266(48.19) | | 4.66 (2.90-7.49) | | < 0.001 |  |
| mRS score of 0–2 | 332(41.45) | 24(18.75) | | 286(51.81) | |  |  |  |  |
| **Secondary**(mRS category) | | | | | | | | |  |
| 0 | 26(3.25) | 1(0.78) | | 25(4.53) | |  | | < 0.001 |  |
| 1 | 125(15.61) | 9(7.03) | | 109(19.75) | |  | |  |  |
| 2 | 181(22.60) | 14(10.94) | | 152(27.54) | |  | |  |  |
| 3 | 218(27.22) | 43(33.59) | | 156(28.26) | |  | |  |  |
| 4 | 183(22.85) | 44(34.38) | | 91(16.49) | |  | |  |  |
| 5 | 43(5.37) | 10(7.81) | | 11(1.99) | |  | |  |  |
| 6 | 25(3.12) | 7(5.47) | | 8(1.45) | |  | |  |  |
| **Primary (6 months)** | **n = 658** | **n = 110** | | **n = 447** | |  | |  |  |
| mRS score of ≥3 | 281(42.71) | 77(70.00) | | 131(29.31) | | 5.63(3.57-8.88) | | < 0.001 |  |
| mRS score of 0–2 | 377(57.29) | 33(30.00) | | 316(70.69) | |  |  |  |  |
| **Secondary** (mRS category) | |  |  |  |  |  |  |  |  |
| 0 | 36(5.47) | 2(1.82) | | 32(7.16) | |  | | < 0.001 |  |
| 1 | 159(24.16) | 10(9.09) | | 138(30.87) | |  | |  |  |
| 2 | 182(27.66) | 21(19.09) | | 146(32.66) | |  | |  |  |
| 3 | 155(23.56) | 50(45.45) | | 82(18.34) | |  | |  |  |
| 4 | 73(11.09) | 14(12.73) | | 34(7.61) | |  | |  |  |
| 5 | 26(3.95) | 6(5.45) | | 7(1.57) | |  | |  |  |
| 6 | 27(4.10) | 7(6.36) | | 8(1.79) | |  | |  |  |

**Table S8**. Analysis subgroup between HAP and SAP

|  | Total | HAP | | SAP | | OR (95%CI) | | P value |
| --- | --- | --- | --- | --- | --- | --- | --- | --- |
| **Discharge** | **n = 922** | **n = 139** | | **n = 157** | |  | |  |
| mRS score of ≥3 | 734(79.61) | 125(89.93) | | 148(94.27) | | 1.84 (0.77-4.41) | | 0.16 |
| mRS score of 0–2 | 188(20.39) | 14(10.07) | | 9(5.73) | |  |  |  |
| **Secondary (**mRS category) | | |  | |  | |  |  |
| 0 | 1(0.11) | 0(0.00) | | 0(0.00) | |  | | **0.017** |
| 1 | 39(4.23) | 1(0.72) | | 2(1.27) | |  | |  |
| 2 | 148(16.05) | 13(9.35) | | 7(4.46) | |  | |  |
| 3 | 257(27.87) | 28(20.14) | | 19(12.10) | |  | |  |
| 4 | 362(39.26) | 68(48.92) | | 72(45.86) | |  | |  |
| 5 | 115(12.47) | 29(20.86) | | 57(36.31) | |  | |  |
| **Primary (3 months)** | **n = 801** | **n = 128** | | **n = 121** | |  | |  |
| mRS score of ≥3 | 469(58.55) | 104(81.2) | | 99(81.8) | | 1.04 (0.55-1.97) | | 0.908 |
| mRS score of 0–2 | 332(41.45) | 24(18.8) | | 22(18.2) | |  |  |  |
| **Secondary (**mRS category) | | | | | | | | |
| 0 | 26(3.25) | 1(0.78) | | 0(0.00) | |  | | 0.016 |
| 1 | 125(15.61) | 9(7.03) | | 7(5.79) | |  | |  |
| 2 | 181(22.60) | 14(10.94) | | 15(12.40) | |  | |  |
| 3 | 218(27.22) | 43(33.59) | | 19(15.70) | |  | |  |
| 4 | 183(22.85) | 44(34.38) | | 48(39.67) | |  | |  |
| 5 | 43(5.37) | 10(7.81) | | 22(18.18) | |  | |  |
| 6 | 25(3.12) | 7(5.47) | | 10(8.26) | |  | |  |
| **Primary (6 months)** | **n = 658** | **n = 110** | | **n = 101** | |  | |  |
| mRS score of ≥3 | 281(42.71) | 77(70) | | 73(72.3) | | 1.12 (0.62-2.03) | | 0.715 |
| mRS score of 0–2 | 377(57.29) | 33(30) | | 28(27.7) | |  |  |  |
| **Secondary (**mRS category) | | | | | | | | |
| 0 | 36(5.47) | 2(1.82) | | 2(1.98) | |  | | 0.007 |
| 1 | 159(24.16) | 10(9.09) | | 11(10.89) | |  | |  |
| 2 | 182(27.66) | 21(19.09) | | 15(14.85) | |  | |  |
| 3 | 155(23.56) | 50(45.45) | | 23(22.77) | |  | |  |
| 4 | 73(11.09) | 14(12.73) | | 25(24.75) | |  | |  |
| 5 | 26(3.95) | 6(5.45) | | 13(12.87) | |  | |  |
| 6 | 27(4.10) | 7(6.36) | | 12(11.88) | |  | |  |

**Table S9**. Multivariable analysis including factor associated with poor outcome (mRS) at discharge

|  | B | S.E. | Sig. | Exp(B) | 95% C.I.for EXP(B) | |
| --- | --- | --- | --- | --- | --- | --- |
|  |  |  |  |  | Lower | Upper |
| > 75 years | 0.24 | 0.13 | 0.061 | 1.27 | 0.99 | 1.63 |
| Male | 0.13 | 0.20 | 0.510 | 1.14 | 0.77 | 1.71 |
| Stroke history | 0.42 | 0.35 | 0.229 | 1.53 | 0.77 | 3.03 |
| Premorbid mRS=0 (baseline) |  |  | 0.828 |  |  |  |
| Premorbid mRS=1 | -0.27 | 0.51 | 0.596 | 0.76 | 0.28 | 2.08 |
| Premorbid mRS=2 | 0.16 | 0.89 | 0.862 | 1.17 | 0.21 | 6.65 |
| Diabetes | -0.07 | 0.23 | 0.751 | 0.93 | 0.60 | 1.45 |
| Atrial fibrillation | 0.89 | 0.55 | 0.103 | 2.44 | 0.84 | 7.10 |
| Hemorrhage | 0.73 | 0.31 | 0.019 | 2.08 | 1.13 | 3.83 |
| Nasogeastric Tube | 0.55 | 0.31 | 0.076 | 1.74 | 0.95 | 3.19 |
| NIHSS_Mild (baseline) |  |  | 0.000 |  |  |  |
| NIHSS_Moderate | 2.15 | 0.20 | 0.000 | 8.56 | 5.79 | 12.64 |
| NIHSS_Severe |  |  |  |  |  | . |
| Early rehabilitation | 0.06 | 0.26 | 0.817 | 1.06 | 0.64 | 1.76 |
| Pneumonia_None (baseline) |  |  | 0.217 |  |  |  |
| Pneumonia_SAP | 0.67 | 0.42 | 0.113 | 1.95 | 0.85 | 4.45 |
| Pneumonia_HAP | 0.31 | 0.36 | 0.389 | 1.37 | 0.67 | 2.78 |
| Disorders of consciousness | 0.76 | 0.45 | 0.089 | 2.15 | 0.89 | 5.17 |

Abbreviation: NIHSS: National Institutes of Health Stroke Scale.

**Table S10**. Multivariable analysis including factor associated with poor outcome (mRS) at 3 months (n=801)

|  | B | S.E. | Sig. | Exp(B) | 95% C.I.for EXP(B) | |
| --- | --- | --- | --- | --- | --- | --- |
|  |  |  |  |  | Lower | Upper |
| > 75 years | 0.18 | 0.11 | 0.092 | 1.20 | 0.97 | 1.48 |
| Male | -0.34 | 0.18 | 0.059 | 0.71 | 0.50 | 1.01 |
| Stroke history | 0.09 | 0.32 | 0.781 | 1.09 | 0.58 | 2.06 |
| Premorbid mRS=0 (baseline) |  |  | 0.854 |  |  |  |
| Premorbid mRS=1 | 0.05 | 0.44 | 0.914 | 1.05 | 0.44 | 2.51 |
| Premorbid mRS=2 | -0.31 | 0.65 | 0.629 | 0.73 | 0.21 | 2.61 |
| Diabetes | 0.17 | 0.20 | 0.388 | 1.19 | 0.81 | 1.74 |
| Atrial fibrillation | -0.13 | 0.39 | 0.732 | 0.88 | 0.41 | 1.88 |
| Hemorrhage | 0.15 | 0.22 | 0.488 | 1.17 | 0.76 | 1.79 |
| Nasogeastric Tube | 0.56 | 0.23 | 0.012 | 1.76 | 1.13 | 2.73 |
| NIHSS_Mild (baseline) |  |  | 0.000 |  |  |  |
| NIHSS_Moderate | 1.75 | 0.20 | 0.000 | 5.75 | 3.86 | 8.56 |
| NIHSS_Severe | 2.36 | 0.51 | 0.000 | 10.55 | 3.87 | 28.76 |
| Early rehabilitation | -0.05 | 0.21 | 0.811 | 0.95 | 0.63 | 1.43 |
| Pneumonia_None (baseline) |  |  | 0.000 |  |  |  |
| Pneumonia_SAP | 0.77 | 0.31 | 0.014 | 2.15 | 1.16 | 3.96 |
| Pneumonia_HAP | 1.10 | 0.29 | 0.000 | 3.00 | 1.70 | 5.29 |
| Disorders of consciousness | 0.62 | 0.31 | 0.043 | 1.86 | 1.02 | 3.38 |

**Table S11**. Multivariable analysis including factor associated with poor outcome (mRS) at 6 months (n=658)

|  | B | S.E. | Sig. | Exp(B) | 95% C.I.for EXP(B) | |
| --- | --- | --- | --- | --- | --- | --- |
|  |  |  |  |  | Lower | Upper |
| > 75 years | 0.25 | 0.12 | 0.040 | 1.28 | 1.01 | 1.62 |
| Male | -0.39 | 0.20 | 0.053 | 0.68 | 0.46 | 1.01 |
| Stroke history | 0.45 | 0.36 | 0.211 | 1.57 | 0.78 | 3.16 |
| Premorbid mRS=0 (baseline) |  |  | 0.526 |  |  |  |
| Premorbid mRS=1 | -0.33 | 0.50 | 0.502 | 0.72 | 0.27 | 1.90 |
| Premorbid mRS=2 | -0.74 | 0.68 | 0.275 | 0.48 | 0.13 | 1.80 |
| Diabetes | 0.20 | 0.22 | 0.369 | 1.22 | 0.79 | 1.90 |
| Atrial fibrillation | 0.29 | 0.43 | 0.509 | 1.33 | 0.57 | 3.12 |
| Hemorrhage | -0.28 | 0.25 | 0.254 | 0.76 | 0.47 | 1.22 |
| Nasogeastric tube | 0.68 | 0.23 | 0.003 | 1.98 | 1.26 | 3.12 |
| NIHSS_Mild (baseline) |  |  | 0.000 |  |  |  |
| NIHSS_Moderate | 1.37 | 0.24 | 0.000 | 3.94 | 2.46 | 6.33 |
| NIHSS_Severe | 2.59 | 0.62 | 0.000 | 13.36 | 3.94 | 45.37 |
| Early rehabilitation | -0.20 | 0.25 | 0.438 | 0.82 | 0.50 | 1.35 |
| Pneumonia_None (baseline) |  |  | 0.000 |  |  |  |
| Pneumonia_SAP | 0.85 | 0.31 | 0.006 | 2.34 | 1.28 | 4.28 |
| Pneumonia_HAP | 1.35 | 0.30 | 0.006 | 3.84 | 2.13 | 6.93 |
| Disorders of consciousness | 1.02 | 0.31 | 0.001 | 2.76 | 1.51 | 5.05 |

**Table S12**: Summary of multivariable analysis of factors associated with poor outcomes (mRS)

|  | At discharge | | | | 3 months | | | | 6 months | | | |
| --- | --- | --- | --- | --- | --- | --- | --- | --- | --- | --- | --- | --- |
|  | Sig. | Exp(B) | 95% C.I. | | Sig. | Exp(B) | 95% C.I. | | Sig. | Exp(B) | 95% C.I. | |
|  |  |  | Lower | Upper |  |  | Lower | Upper |  |  | Lower | Upper |
| > 75 years | 0.061 | 1.27 | 0.99 | 1.63 | 0.092 | 1.20 | 0.97 | 1.48 | 0.040 | 1.28 | 1.01 | 1.62 |
| Male | 0.510 | 1.14 | 0.77 | 1.71 | 0.059 | 0.71 | 0.50 | 1.01 | 0.053 | 0.68 | 0.46 | 1.01 |
| Stroke history | 0.229 | 1.53 | 0.77 | 3.03 | 0.781 | 1.09 | 0.58 | 2.06 | 0.211 | 1.57 | 0.78 | 3.16 |
| Premorbid mRS | 0.828 |  |  |  | 0.854 |  |  |  | 0.526 |  |  |  |
| 1 | 0.596 | 0.76 | 0.28 | 2.08 | 0.914 | 1.05 | 0.44 | 2.51 | 0.502 | 0.72 | 0.27 | 1.90 |
| 2 | 0.862 | 1.17 | 0.21 | 6.65 | 0.629 | 0.73 | 0.21 | 2.61 | 0.275 | 0.48 | 0.13 | 1.80 |
| Diabetes | 0.751 | 0.93 | 0.60 | 1.45 | 0.388 | 1.19 | 0.81 | 1.74 | 0.369 | 1.22 | 0.79 | 1.90 |
| Atrial fibrillation | 0.103 | 2.44 | 0.84 | 7.10 | 0.732 | 0.88 | 0.41 | 1.88 | 0.509 | 1.33 | 0.57 | 3.12 |
| Hemorrhage | 0.019 | 2.08 | 1.13 | 3.83 | 0.488 | 1.17 | 0.76 | 1.79 | 0.254 | 0.76 | 0.47 | 1.22 |
| Nasogeastric Tube | 0.076 | 1.74 | 0.95 | 3.19 | 0.012 | 1.76 | 1.13 | 2.73 | 0.003 | 1.98 | 1.26 | 3.12 |
| NIHSS | 0.000 |  |  |  | 0.000 |  |  |  | 0.000 |  |  |  |
| Moderate | 0.000 | 8.56 | 5.79 | 12.64 | 0.000 | 5.75 | 3.86 | 8.56 | 0.000 | 3.94 | 2.46 | 6.33 |
| Severe |  |  |  | . | 0.000 | 10.55 | 3.87 | 28.76 | 0.000 | 13.36 | 3.94 | 45.37 |
| Early rehabilitation | 0.817 | 1.06 | 0.64 | 1.76 | 0.811 | 0.95 | 0.63 | 1.43 | 0.438 | 0.82 | 0.50 | 1.35 |
| Pneumonia | 0.217 |  |  |  | 0.000 |  |  |  | 0.000 |  |  |  |
| SAP | 0.113 | 1.95 | 0.85 | 4.45 | 0.014 | 2.15 | 1.16 | 3.96 | 0.006 | 2.34 | 1.28 | 4.28 |
| HAP | 0.389 | 1.37 | 0.67 | 2.78 | 0.000 | 3.00 | 1.70 | 5.29 | 0.000 | 3.84 | 2.13 | 6.93 |
| Disorders of consciousness | 0.089 | 2.15 | 0.89 | 5.17 | 0.043 | 1.86 | 1.02 | 3.38 | 0.001 | 2.76 | 1.51 | 5.05 |
